# “Free won’t” after a beer or two: chronic and acute effects of alcohol on neural and behavioral indices of intentional inhibition

**DOI:** 10.1186/s40359-019-0367-z

**Published:** 2020-01-07

**Authors:** Yang Liu, Wery P. M. van den Wildenberg, Gorka Fraga González, Davide Rigoni, Marcel Brass, Reinout W. Wiers, K. Richard Ridderinkhof

**Affiliations:** 1 Department of Psychology, University of Amsterdam, Nieuwe Achtergracht 129B, 1018 WS Amsterdam, The Netherlands; 2 Addiction, Development, and Psychopathology (ADAPT) Lab, Department of Psychology, University of Amsterdam, Amsterdam, The Netherlands; 3 Amsterdam Brain & Cognition (ABC), University of Amsterdam, Amsterdam, The Netherlands; 4 Department of Developmental Psychology, University of Zurich, Zurich, Switzerland; 5 Department of Experimental Psychology, Ghent University, Ghent, Belgium

**Keywords:** Intentional inhibition, Alcohol use, Electroencephalography, Readiness potential, Stop-signal task, Chasing memo task

## Abstract

**Background:**

Response inhibition can be classified into stimulus-driven inhibition and intentional inhibition based on the degree of endogenous volition involved. In the past decades, abundant research efforts to study the effects of alcohol on inhibition have focused exclusively on stimulus-driven inhibition. The novel Chasing Memo task measures stimulus-driven and intentional inhibition within the same paradigm. Combined with the stop-signal task, we investigated how alcohol use affects behavioral and psychophysiological correlates of intentional inhibition, as well as stimulus-driven inhibition.

**Methods:**

Experiment I focused on intentional inhibition and stimulus-driven inhibition in relation to past-year alcohol use. The Chasing Memo task, the stop-signal task, and questionnaires related to substance use and impulsivity were administered to 60 undergraduate students (18–25 years old). Experiment II focused on behavioral and neural correlates acute alcohol use on performance on the Chasing Memo task by means of electroencephalography (EEG). Sixteen young male adults (21–28 years old) performed the Chasing Memo task once under placebo and once under the influence of alcohol (blood alcohol concentration around 0.05%), while EEG was recorded.

**Results:**

In experiment I, AUDIT (Alcohol Use Disorder Identification Test) total score did not significantly predict stimulus-driven inhibition or intentional inhibition performance. In experiment II, the placebo condition and the alcohol condition were comparable in terms of behavioral indices of stimulus-driven inhibition and intentional inhibition as well as task-related EEG patterns. Interestingly, a slow negative *readiness potential* (RP) was observed with an onset of about 1.2 s, exclusively before participants stopped intentionally.

**Conclusions:**

These findings suggest that both past-year increases in risky alcohol consumption and moderate acute alcohol use have limited effects on stimulus-driven inhibition and intentional inhibition. These conclusions cannot be generalized to alcohol use disorder and high intoxication levels. The RP might reflect processes involved in the formation of an intention in general.

## Background

Imagine having cocktails with friends at a bar during happy hour time, and experiencing a strong urge to order one more. But then you realize that you need to prepare for an important meeting the next morning and you decide to refrain from having another drink. In examples like this, there is no external cue signaling a brake, yet you voluntarily suppress your urge for the sake of other priorities. Here, we refer to this type of cognitive control as intentional inhibition. In the current study, we will investigate how intentional inhibition 1) is associated with typical alcohol use and 2) affected by acute alcohol consumption.

## Alcohol use and inhibition

Inhibitory control is defined as the ability to control one’s attention, behavior, thoughts, and/or emotions and instead do what is more appropriate or needed [1]. This ability enables us to override strong internal predispositions or external lures, and do what is more appropriate or needed. Long-term alcohol use has been associated with structural as well as functional neural deficits that are related to inhibition [2]. For instance, alcohol-dependent patients show selective deficits in prefrontal gray and white matter volume [3]; compared to light drinkers, heavy drinkers were slower to stop inappropriate responses and showed deviant amplitudes of the P3 (a brain potential that correlates with the efficiency of response inhibition) [4]. Despite relatively robust neurological evidence for inhibition deficits, alcohol use severity is not consistently associated with impaired behavioral performance of response inhibition [5–7]. Acute alcohol use (moderate to high dosage), by contrast, was more consistently related with inhibition deficits [8, 9] and reduced amplitudes of inhibition-related brain potentials [10].

## Intentional inhibition

Theoretically, motor inhibition can be classified into stimulus-driven inhibition and intentional inhibition based on the degree of endogenous volition involved [11]. A daily-life example of stimulus-driven inhibition is stopping to a traffic-light that suddenly turns to red. The past decades have seen abundant research efforts exclusively into the effects of alcohol on stimulus-driven inhibition (see reviews: [12–14]). However, rather than relying on external cues, deciding independently when and/or whether to abort an action plays an even more important role in daily life [15]. Intentional inhibition refers to the capacity to voluntarily suspend or inhibit an about-to-be-executed action at the last moment [16]. In terms of drinking, the priming dose effect of alcohol, i.e., loss of control over further consumption after a priming dosage, reflects the insufficiency of intentional inhibition rather than stimulus-driven inhibition [17].

There have been several attempts to study intentional inhibition using varieties of the Libet task [18], the Marble Task [19], and the modified go/no-go task [20, 21]. To investigate intentional inhibition, these tasks usually included a free-choice condition, where participants were encouraged to act/inhibit voluntarily and roughly equally across all the trials. For instance, in the Marble task, participants view a white marble rolling down a ramp. In 50% of the trials, the marble turns green and participants have to stop it from crashing as fast as possible by pressing the button. If the marble remains white, the participants can choose between performing the prepared action (i.e., stop the marble) and execution of intentional inhibition (i.e., do not stop the marble). Such “free choice” design is suboptimal in at least three ways regarding the concept of intentional inhibition. First, the choice between acting and withholding is relatively arbitrary; little (if anything) really hinges on whether the participant decides to act or not on any particular trial. Accordingly, participants might behave in a way that they believe will satisfy the experimenters’ definition of volition. Second, participants are subject to substantial time pressure, which may prevent the time-consuming development of spontaneous intentions. Third, participants may pre-decide on whether and when to inhibit ahead of time (even before the start of the trial) rather than on the fly [22], even when emphasizing that this is to be avoided. Thus, the study of intentional inhibition may be augmented by using more ecologically valid tasks.

## The present study

To address these points, a novel task was developed, in which stimulus-driven and intentional inhibition can be measured under comparable conditions that are ecologically more representative (Rigoni, Brass, van den Wildenberg, & Ridderinkhof, *unpublished manuscript*). In the current study, we will investigate if and how alcohol use affects intentional inhibition in two complementary ways. Experiment I focuses on prolonged (i.e., last year) alcohol use in relation to intentional versus stimulus-driven inhibition with a relatively large sample. The Chasing Memo task, as well as the classic stop-signal task (SST), were administered. Experiment II investigates the behavioral and neural effects of acute alcohol use on the Chasing Memo task performance. Electroencephalographic (EEG) activity was recorded in a smaller sample, with a double-blind, placebo-controlled, within-subject design.

## Experiment I

### Introduction

The aim of the Experiment I was to test whether past-year typical alcohol use influenced stimulus-driven as well as intentional inhibition. Extensive research into the effects of long-term alcohol use on stimulus-driven inhibition has been documented, but the conclusions are inconsistent. Some researchers found that compared to controls, heavy drinkers showed impaired stopping performance, signified by either longer stop-signal reaction time (SSRT) on the SST [4] or higher commission error rates in the go/no-go task (GNG) [23, 24]. These findings, however, conflict with a series of other studies. For instance, a meta-analysis of differences between heavy drinkers and controls reported null-effects with respect to inhibitory impairments in 9 out of 12 GNG studies and in 7 out of 9 studies using the SST [13]. Similarly, in a recent retrospective epidemiological study among 2230 adolescents, longitudinal analyses showed that 4 years of weekly heavy drinking did not result in impairments in basic executive function, including inhibitory control [25].

In the literature, two types of impulsivity have been discerned that may trigger failures of inhibitory control: ‘stopping impulsivity’ and ‘waiting impulsivity’, which rest on largely distinct neural circuits [26, 27]. ‘Stopping impulsivity’ refers to impairments in the ability to interrupt an already initiated action, whereas ‘waiting impulsivity’ refers to impairments in the ability to refrain from responding until sufficient information has been gathered or a waiting interval has elapsed. Stopping and waiting impulsivity have typically been tested in the SST and in the delay discounting task, respectively [28]. In the Chasing Memo task (Rigoni et al., *unpublished manuscript*), participants were asked to use the computer mouse to move the cursor and chase a small fish, called Memo, as it moves across the screen (“swimming” against a nautical background picture). Participants disengaged from visuomotor tracking in response to either an external stop cue (i.e., stimulus-driven inhibition) or at will (i.e., intentional inhibition).

Meanwhile, to supplement and validate the stimulus-driven inhibition component of the new task, the conventional SST was also administered [29]. In addition to laboratory-based tasks, two sets of questionnaires were also administered. The Barratt Impulsiveness Scale (BIS-11) [30], and Dickman’s Impulsivity Inventory (DII) [31], were used to test impulsivity. Substance use was tested by the AUDIT (Alcohol Use Disorder Identification Test) [32], the mFTQ (modified version of the Fagerström tolerance questionnaire) [33], the CUDIT-R (cannabis use disorder identification test revised) [34], and the CORE (the core alcohol and drug survey) [35].

The current study focuses on college students, for whom alcohol is one of the most frequently used substances, and it gives rise to unsafe drinking-&-driving behavior and the consumption of other substances [36]. Although prior work (as reviewed above) has not yielded consistent results, we tested the hypothesis that higher AUDIT scores (i.e., more risky alcohol use within the past 12 months) were associated with prolonged SSRTs (analogous to longer disengage latencies in the cued version of the Chasing Memo task). For intentional inhibition in the Chasing Memo task, we conceived of two opposing scenarios: analogous to stimulus-driven inhibition, past-year alcohol use induces ‘stopping impulsivity’ and delays intentional disengagement; alternatively, it induces ‘waiting impulsivity’ and results in faster disengagement times [27]. Although the lack of existing studies on alcohol and intentional inhibition prevents us from inferring strong theory-based hypotheses, the present task set-up will allow us to empirically distinguish between them.

### Methods^1^

#### Participants

Eighty-six undergraduate students (10 males) were recruited (age: *Mean* = 20.77, *SD* = 1.86). Inclusion criteria included: 1) between 18 and 25 years old; 2) no report of head injuries, colorblindness or seizures; 3) no prior and current diagnosis of depression; 4) proper mastery of Dutch, as all task instructions and questionnaires were shown in Dutch. Due to incorrect settings of refresh rates on some test computers, we cannot use the Chasing Memo data from a subset of 26 participants.^2^ Thus, the analyses of the Chasing Memo task were based on the remaining 60 subjects (6 males, 20.75 ± 2.01 years old).

#### Questionnaires

The BIS-11 is a 30-item questionnaire designed to assess the personality/behavioral construct of impulsiveness [30]. The DII included two subscales: functional impulsivity (11 items) and dysfunctional impulsivity (12 items). The AUDIT is a 10-item survey used as a screening instrument for excessive or hazardous alcohol use [32]. It covers the domains of recent alcohol consumption (items 1–3), alcohol dependence symptoms (items 4–7), and alcohol-related problems (items 8–10). The mFTQ assesses the level of nicotine dependence among adolescents [33]. The CUDIT-R was used to identify individuals who have used cannabis in problematic or harmful ways during the preceding 6 months [34]. The CORE was originally designed to examine the use, scope, and consequences of alcohol and other drugs in the college settings [35]. In the current research, participants were asked to indicate how often within the last year and month they had used each of the 11 types of drugs. Reliability of these questionnaires can be found in Additional file 1.

#### Behavioral tasks

##### Chasing memo task

**Fig. 1:**
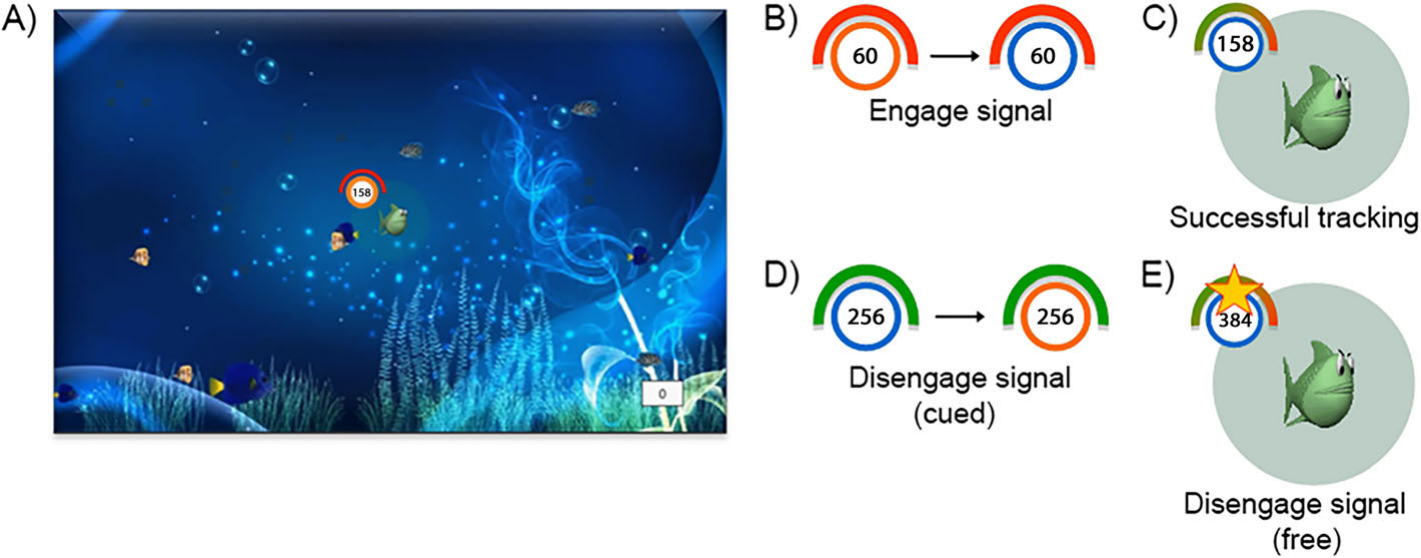
The Chasing Memo Task. **a** Background display for the motor tracking task. Participants were instructed to track fish Memo around the screen by keeping the mouse within the green zone surrounding the target. On each trial, a counter was displayed on the bottom right of the screen which displayed the points earned during successful tracking; **b** When the circle turned from orange to blue, participants started tracking either at will (intentional condition) or as quickly as possible (cued condition); **c** During successful tracking, the half-circle red bar gradually turned green, signaling that the participant started to earn points; **d** In the cued condition, the circle switched back to orange to signal that the participant has to stop tracking as quickly as possible; **e** In the intentional condition, the appearance of a star indicated the beginning of a time window in which the participant can earn additional bonus points. In these trials, participants can decide voluntarily when to disengage from motor tracking in order to collect the bonus points

In this task, an animated fish called Memo is moving (‘swimming’) at 360 pixels/sec against the background of the bottom of an ocean, changing directions at random angles between 0 and 115 degrees, at intervals between 556 and 1250 ms. The participants’ main task was to track the fish by keeping a yellow dot (operated through the computer mouse) within close proximity of Memo (i.e., within a green zone of 2 cm radius surrounding it). Points were earned per second during successful tracking (i.e., as long as the cursor is within this green zone) and accumulated points were displayed in the bottom right corner of the screen (tracking points). These points accumulated faster as a linear function of time spent within the green proximity zone. Accumulation rate was indicated to the subject by a red/green bar, which turned from red to green as a function of accurate tracking (see Fig. 1). Upon failures to chase Memo (i.e., failing to keep the yellow dot within the green zone), accumulation rates were reset, and accumulation of points would again start slowly as soon as the participant resumed successful tracking and then rise as a function of accurate tracking time. Participants were told that tracking points were converted to real money, which can yield up to 5 euro extra at the end of the experiment. Thus, participants had a strong immediate incentive motivation to continue accurate tracking.

A circle at the top left corner of the green zone served as the external signal to start and stop tracking. At the beginning of the trial, the circle was colored orange; after a variable delay (between 3 and 6 s) it turned blue (*go signal*), indicating that participants can start tracking the target. The specific instructions differed depending on the experimental condition.

*In the cued condition,* participants were instructed to start tracking as fast as possible when the *go signal* appeared (cued engagement) and stop as soon as possible if the circle turned orange again, i.e., the *stop signal* (cued disengagement). Participants were asked to disengage by leaving the mouse completely still in its end position. The trial ended 2 s after tracking disengagement. Within the colored circle, there was a counter with a serial display of digits constituting a number (between 100 and 999). Every 100 ms, that number incremented by 1 until the value of 999 was reached, after which the counter was reset to 100. Participants had to remember the number when the *stop cue* appeared and type in the number by the end of a trial and how confident they were about their answers (from 1 to 7). This is used as the timing accuracy index.

*In the free condition,* participants can freely decide when to start tracking after the *go signal* appeared. After uninterrupted successful tracking for 2 s, a *bonus signal*, signified by a yellow star, was displayed next to the red/green meter (Fig. 1). Its appearance signaled the beginning of a 20 s (participants did not know the length) temporal window within which participants were to continue tracking until they felt the urge to stop. Disengagement meant foregoing the immediate reward (increase in normal points) in favor of the future reward (bonus points). The number of bonus points varied between 2 and 50 and was determined by the disengagement moment. Participants were instructed that some variability in their tracking latency (within the margins of not stopping too soon nor too late) would benefit an optimal amount of bonus points. Unbeknownst to the participants, the time at which the star was lost was determined stochastically by drawing randomly from a normal distribution, such that the optimum waiting time was 10 s on average; prolonged tracking would be highly beneficial on some trials but highly detrimental on others. Within each block of the *free condition*, bonus points were accumulated across trials and converted into extra time (1 second per earned bonus point) for tracking in a later *bonus trial*. In a *bonus trial*, participants can earn tracking-points 4 times as fast as that in a regular trial. Thus, more bonus points result in a higher total of tracking-points (and hence in greater earnings). In order to prevent undesirable response tendencies, participants were instructed and trained to follow their urge rather than preplan their time of disengagement or use external cues (such as spatial position or counter value) to determine the time of disengagement. As in the *cued condition*, participants now had to register and report the number of this counter at the time they first felt the urge (or conscious intention) to disengage, i.e., the W-moment [38].

Detailed instructions were provided at the beginning of the experiment, and participants performed a guided practice session to familiarize them with the task. The entire experimental session consisted of 6 cued and 6 free blocks of 10 trials each. Cued and free blocks were presented in alternating order and every free block was followed by a bonus trial.

##### SST

Similar to the task used by van den Wildenberg et al., (2006), participants were required to respond quickly and accurately with the corresponding index finger to the direction of a right- or a left-pointing green arrow (*go trials*). Arrow presentation was response-terminated. The green arrow changed to red on 25% of the trials (*stop trials*), upon which the go response had to be aborted. Intervals between subsequent go signals varied randomly but equiprobably, from 1750 to 2250 ms in steps of 50 ms, where a black fixation point (10 × 10 pixels) was presented. A staircase-tracking procedure dynamically adjusted the delay between the onset of the go signal and the onset of the stop signal (SSD) for each hand separately to control inhibition probability [39]. SSD started at 100 ms and increased by 50 ms after a successful inhibition, and decreased by 50 ms after a failed inhibition. The SST consisted of five blocks of 60 trials, the first of which served as a practice block to obtain stable performance [29]. The SST measures both the efficiency of response execution (mean reaction time to correct go-signals, go RT) and the latency of stimulus-driven inhibitory control (SSRT), where longer SSRT reflects a general slowing of inhibitory processes [40]. The integration method was used for SSRT calculation [41, 42].

#### Procedure

All participants signed informed consent prior to the laboratory session. They performed two computer tasks in a counterbalanced sequence, with a series of questionnaires in between, and the behavioral tasks were administered using Presentation® software [43]. The procedures were approved by the local ethics committee and complied with institutional guidelines and the declaration of Helsinki. Participants were rewarded either €15 or 1.5 credit points upon accomplishment.

#### Data preparation and statistical analysis

##### Chasing memo task

Although Disengage RT was our measurement of primary interest, Engage RT was also analyzed to verify whether chronic alcohol use affected basic response speed. Engage RT (the time from the engage color change until the start of tracking) was calculated for both cued and free conditions. Engage RTs less than 100 ms were discarded from the analysis, resulting in 3360 (93.3%) out of 3600 trials for the cued condition and 3381 (93.9%) for the free condition. Disengage RT in the cued condition was calculated by subtracting the time of the disengage color change from the time at which tracking was completely halted. For the free condition, Disengage RT is the time from the appearance of the bonus star until the time of arrested tracking. Before analysis, 376 (10.4%) trials in the free condition were removed as intentional inhibition failures, i.e., participants did not stop tracking within the provided time window (20 s).

The W-interval in the free condition was computed as the interval between the reported W-moment until the time of the actual stopping. In the cued condition, timing accuracy was the difference between the reported and the actual appearance moment of the stop signal.

For all RT-related dependent variables, the median rather than mean value was used for further analysis as RT distributions were not normally distributed for all of the participants (skewed to the left for some participants and to the right for others). Engage RT and Disengage RT were analyzed using multiple linear regressions with AUDIT sum score^3^ (AUDIT sum was nearly normally distributed with Skewness of 0.06 and Kurtosis of − 0.68) and Inhibition Category (free vs. cued) as predictors, controlling for gender.^4^ The possible association between past-year alcohol use and timing accuracy was examined by Pearson correlation. W-interval was analyzed with AUDIT score as a predictor and controlled for timing accuracy. These analyses were performed using SPSS 24.0 [45].

##### SST

The successful inhibition percentages on inhibition trials ranged from 28.3 to 63.3% (*M* = 49.6%, *SD* = 4.67%), which meets the requirements of the integration method for SSRT calculation [41]. To compute go RT, only correct responses were taken into account. Afterward, similar regression analyses as the Chasing Memo task was performed for SSRT and go RT separately without the factor of Inhibition Category. We analyzed data once with all the participants (*N* = 86) and once with those also had Chasing Memo task performance (*N* = 60).

In addition, two correlation matrices were built: 1) correlations between different substances use; 2) correlations between different measures of impulsivity (Disengage RT in the free condition, SSRT, BIS-11 score, and DII score).

##### Combination of conventional and Bayesian-based analysis

To quantify the strength of our findings beyond standard significance testing and to remedy the relatively small sample size caused by the technical failure, the main hypotheses were also examined by calculating a Bayes Factor using Bayesian Information Criteria [46–49]. The Bayes factor provides the odds ratio (BF_01_) for the null versus the alternative hypotheses given a particular data set (BF_10_ is simply the inverse of BF_01_). A value of 1 means that the null and alternative hypotheses are equally likely; values larger than 1 suggest that the data are in favor of the null hypothesis, and values smaller than 1 indicate that the data are in favor of the alternative hypothesis. A BF_01_ between 1 and 3 indicates anecdotal evidence for the null compared to an alternative hypothesis, 3–10 indicates moderate evidence and 10–30 indicates strong evidence [50, 51]. The BFs were calculated with JASP 0.9.2.0., an open-source statistical package [52].

### Results

#### Sample characteristics

Descriptive statistics (i.e., mean, standard deviation, minimum and maximum values) of the tested variables (demographics, substance use, task performance, and trait impulsivity) can be found in Table 1.

**Table 1:** Descriptive statistics for substance use, task performance and trait impulsivity

		Substance Use					SST			Chasing Memo task^2^						Trait Impulsivity					
	Age	AUDIT	CUDIT	Fagerstroim	CORE^1^/Last Year	CORE/Last Month	go RT	SSRT	stop rate	Disengage RT		Engage RT		W interval	Timing Accuracy	BIS attention	BIS motor	BIS non-planning	BIS total	DII dysfunctional	DII functional
										cued	free	cued	free								
Min.	18	0	0	0	0	0	327.7	90	28.33	353	1017	304.5	205	−526	16.67	9	13	13	40	1	2
Max.	25	23	24	6	320	140	1014	272.3	63.33	3793	15,157	756	722.5	7749	5168	30	33	33	81	10	9
Mean	20.75	10.07	2.5	1.53	90.25	25.25	441.3	197.8	49.67	748.7	8662	410.7	407.6	528.7	852.4	17.25	20.3	23.25	60.8	5.42	5.87
SD	2.01	5.6	4.33	1.39	84.79	39.46	135.9	33.87	4.65	583.6	3619	86.34	76	1233	932.5	3.86	4.07	4.1	9.25	2.16	1.49

*SST* stop-signal task

^1^Sum score of CORE excluding alcohol use

^2^Mean and SD for RT-related variables in the Chasing Memo task were calculated based on median values

#### Chasing memo task

Task difficulty was assessed by the number of times one lost the star. Out of the 120 trials, on average participants lost the star 31 times (*SD* = 21), ranging from 6 to 145. This indicates that most of the participants have a good mastery of the task and should be able to allocate attention to their behavioral intentions.

Variables used in the regression analyses were checked for multicollinearity using variance inflation factors (VIF) before being entered into the multivariate analyses; VIF for all variables were below 2 for the following regression models. The linear regression model for Engage RT was not significant (*F* (3, 116) = 0.99, *p =* 0.39), with a *R*^*2*^ of 0.025. None of the explanatory variables significantly predicted Engage RT (AUDIT: *β =* 0.10*, p =* 0.29; Inhibition Category: *β =* − 0.02*, p =* 0.84; gender: *β =* − 0.12*, p =* 0.19). Bayesian linear regression showed that the null model provided a fit that was 2.2 times better than the model that added the factor gender, 3.0 times better than the model that added AUDIT and 5.1 times better than the model that added Inhibition Category.

The linear regression model for Disengage RT was significant (*F* (3, 116) = 94.48*, p* < 0.01), with a *R*^*2*^ of 0.71. Inhibition Category significantly predicted Disengage RT (*β =* 0.84*, p* < 0.01). Disengage RT was much longer in the free condition than in the stimulus-driven inhibition (8662 ms vs. 749 ms). Neither AUDIT (*β =* − 0.06*, p =* 0.27) nor gender (*β =* 0.06*, p =* 0.27) predicted Disengage RT. Bayes factor analysis confirmed this by showing that the model with factor Inhibition Category provided a fit that was 7.0 times and 7.2 times better than the model that further added factor Gender and AUDIT, respectively.

Past-year risky alcohol consumption is not associated with alteration in timing accuracy (*r* = − 0.21, *p* = 0.10, BF_01_ = 1.66). The linear regression model for W-interval was not significant (*F* (2, 57) = 0.14, *p =* 0.87), with a *R*^*2*^ of 0.005. None of the explanatory variables significantly predicted W-interval (AUDIT: *β =* − 0.007*, p =* 0.96; timing accuracy: *β =* − 0.071*, p =* 0.60). Bayes factor analysis confirmed this by showing that the null model provided a fit that was 3.4 times, and 3.8 times better than the model that added the factor Timing Accuracy and AUDIT, respectively.

#### SST

There were no qualitative differences between the outcomes with different sample size (86 vs. 60). We report the results for the smaller sample size (same as the Chasing Memo task) below, and the larger sample size in Additional file 1. The linear regression model for SSRT was not significant (*F* (2, 57) = 0.47, *p =* 0.63), with a *R*^*2*^ of 0.02. None of the explanatory variables significantly predicted SSRT (AUDIT: *β =* 0.11*, p =* 0.43; gender: *β =* 0.07*, p =* 0.58). Bayes factor analysis confirmed this by showing that the null model provided a fit that was 2.9 times, and 3.4 times better than the model that added the factor AUDIT and Gender, respectively. The linear regression model for go RT was not significant either (*F* (2, 57) = 2.40, *p =* 0.10), with a *R*^*2*^ of 0.078. AUDIT was a significant predictor of go RT (*β =* − 2.68*, p =* 0.04), indicating the higher the AUDIT score the shorter the go RT. Gender was not a strong predictor of go RT (*β =* − 0.08*, p =* 0.52). Bayes factor analysis indicated anecdotal evidence for the effect of AUDIT, i.e., adding it to the model was just 1.6 times better than the null model. And the fitness of the null model is 3.3 times better than adding factor Gender.

Results were very similar when AUDIT-C was used (see Additional file 1).

#### Correlation matrix

As was shown in Table 2, alcohol use and other substances use (e.g., cigarette and cannabis use) were highly correlated, which can be expected. In Table 3, the correlation matrix revealed three significant correlations between different impulsivity measures. SSRT correlated negatively with the attentional subscale of BIS-11 (*r* = − 0.20, *p* = 0.03, BF_10_ = 1275), and correlated positively with the motor subscale of BIS-11 (*r* = 0.22, *p* = 0.01, BF_10_ = 2122). In addition, the motor subscale of BIS-11 and the dysfunctional subscale of DII were negatively correlated (*r* = − 0.21, *p* = 0.02, BF_10_ = 1395). Subscales of impulsivity, either measure by BIS-11 or DII were not correlated with Chasing Memo task performance.^5^

**Table 2:** Correlation matrix between substance use

			1	2	3	4
1	AUDIT	r				
		BF_10_				
2	CUDIT	r	0.27**			
		BF_10_	8296.00			
3	Fagerström	r	0.32**	0.41**		
		BF_10_	30,554.00	1593.00		
4	CORE/last month	r	0.30**	0.41**	0.70**	
		BF_10_	24,639.00	5988.00	6.436e + 14	
5	CORE/last year	r	0.61**	0.48**	0.64**	0.73**
		BF_10_	7.271e + 10	368,020.00	1.679e + 11	3.534e + 18

* p < 0.05, ** p < 0.01

**Table 3:** Correlation matrix between impulsivity measures

			1	2	3	4	5	6	7
1	Disengage RT (free)	r							
		BF_10_							
2	Disengage RT (cued)	r	−0.17						
		BF_10_	0.38						
3	SSRT	r	0.02	0					
		BF_10_	0.16	0.16					
4	BIS attentional	r	0.06	−0.05	−0.20*				
		BF_10_	0.18	0.17	1275				
5	BIS motor	r	0.17	0.12	0.22*	0.27**			
		BF_10_	0.37	0.25	2122	9251			
6	BIS non-planning	r	0.06	0.15	0.09	0.30**	0.58*		
		BF_10_	0.18	0.31	0.18	26,303	1.863 × 10^9^		
7	DII dysfunctional	r	−0.09	−0.16	0.17	0.04	−0.21*	−0.11	
		BF_10_	0.2	0.33	0.68	0.12	1395	0.22	
8	DII functional	r	0.09	−0.03	−0.1	−0.15	−0.04	− 0.14	−0.41**
		BF_10_	0.206	0.165	0.206	0.43	0.126	0.353	4859

* p < 0.05, ** p < 0.01

### Discussion

In the first experiment, a past-year increase in risky drinking showed no relationship with any of the inhibition-related tasks and questionnaires. In the SST, alcohol use slightly speeded response latency, but had no influence on the inhibition process. In the Chasing Memo task, typical alcohol use hardly had any effect on Engage RT and Disengage RT, nor did it influence the W-interval. The correlation analysis confirmed the existence of polysubstance use and the multidimensional feature of impulsivity (i.e., impulsivity measures are not largely correlated).

#### Stimulus-driven inhibition

Our findings on stimulus-driven inhibition were comparable between the Chasing Memo task and the standard SST. For stimulus-driven inhibition as tested by the SST, the present null findings of past-year alcohol use are replications of some recent studies [25, 53], but conflicted with some others [13]. Against the backdrop of the fairly inconsistent literature, it’s time to re-assess the connection between recreational moderate alcohol use and stimulus-driven inhibition impairment. In the current study, alcohol use was regarded as a continuous variable, which allowed drawing conclusions from a relatively complete population. Relatedly, in our recent individual-level mega-analysis, very limited evidence supporting such deteriorating relationship was found across a broad range of substances [54]. As only a small proportion of the participants are diagnosed with Substance Use Disorder (SUD), it is still unclear whether these conclusions would also apply to SUD. By contrast, the so-called *extreme group design*s were frequently used in this field, e.g., comparing light/non-drinkers versus people with alcohol use disorder (AUD) [55]. Studies with such designs yielded more positive findings [56, 57]. Seemingly, people located at the very right end of the continuum, i.e., those diagnosed with alcohol use disorder indeed have difficulties in inhibition. But it does not necessarily mean these findings can be generalized readily to the majority who drink alcohol on a regular/non-hazardous basis, at least on the behavioral level [58].

#### Intentional inhibition

Given that this was the first attempt, we did not have firm a priori predictions on the presence and direction of effects of alcohol use on intentional inhibition. At least in the current context, there was no clear effect of alcohol use on intentional inhibition. The latency of intentional inhibition was expressed by the Disengage RT in the free condition. Its histogram for each individual either showed a rectangle or approximately normal (with mean of near 10 s) distribution, which confirms the validity of the manipulation, in the sense that strategies other than ‘following one’s urge’ (such as counting or waiting strategies) would have resulted in heavily peaked and/or skewed distributions. Though in the free condition participants appeared to start tracking as soon as possible, this did not invalidate the operationalization. As Engagement is less of our focus, we did not emphasize the ‘free will’ as much as for the Disengagement. Also, no consequences were associated with the engage response pattern.

For the W-interval, participants reported to consciously feel the urge to stop about half a second before the actual disengagement. The W-interval was similar for both groups. In the Libet task, the W-moment was reported 200 ms before intentional action [38]. This difference in timing might be due to the dissimilarity between voluntary action and voluntary inhibition, as well as specific task features, which will require further investigation.

Although some limitations may apply, the consistency of effects and the robustness of the evidence in favor of the null hypotheses (as confirmed by Bayesian analyses) appears to justify the conclusion that a limited period (i.e., 1 year or a bit longer) of heavy drinking does not affect intentional or stimulus-driven inhibition (at least not in university students). However, before accepting such a conclusion, we seek further evidence through adopting a manipulation that in past research has proven more potent in inducing alcohol-related effects on stimulus-driven inhibition. Alcohol use may increase maladaptive behaviors either because of lasting sequelae of chronic use or through its direct, acute effects [59]. Acutely, alcohol may impair cue-based inhibition and result in an increased likelihood of engaging in risky behaviors, such as driving while intoxicated. In addition, alcohol-induced impairments may also affect the likelihood of further unplanned consumption of alcohol [60]. Several laboratory studies showed that a moderate acute dosage of alcohol use leads to impaired inhibition on GNG and SST [61, 62]. Therefore, as a next step, we explored if alcohol intoxication affects stimulus-driven and intentional inhibition. In addition to behavioral measures, we also used EEG to record neural activity. This may reveal the acute effects of alcohol on information processing that remain hidden when focusing on behavioral outcomes. For example, EEG highlighted the nature of the effects of alcohol consumption (vs. placebo) on performance monitoring and error correction [63]. Likewise, EEG signals have reflected differences between alcohol effects in light versus heavy drinkers in the absence of differences in behavior [10, 64, 65].

## Experiment II

### Introduction

The aim of Experiment II was to test whether and how acute alcohol use influences intentional inhibition. Compared to chronic alcohol use, acute alcohol administration was more consistently related to impaired stimulus-driven inhibition [66–71]. By analogy, acute alcohol administration might also be more likely to influence intentional inhibition than chronic alcohol use. Loss-of-control over drinking depicts the phenomenon that small to moderate amount of alcohol use induces physical demand/craving for further drink and promotes alcohol-seeking behavior [17, 72, 73]. In this way, people are likely to fail in intentional inhibition and drink more than planned on a typical drinking occasion.

If alcohol affects intentional inhibition, it may affect not only the time of overt disengagement but also the temporal unfolding of that intention. With its unique temporal resolution, EEG may provide a useful candidate study tool for this purpose. The EEG component we are interested in is the readiness potential (RP) or Bereitschaftspotential. It was first recorded by Kornhuber and Deecke (1964) and attracted broad attention after Libet and colleagues’ striking work in 1983 [38, 74]. In their experiment, participants were instructed to press a response button whenever they became aware of the intention to do so and report the time of this urge (the W-moment). They found that the W-moment occurred some 200 ms prior to actual action and about 500 ms after the RP onset [38]. This finding was explained as the brain decides to initiate certain actions prior to any reportable subjective awareness, which raised perhaps unprecedented discussion in the literature. It was recently claimed that the RP might neither give rise to the W-moment (conscious intention) nor to the voluntary movement, as the RP occurs 1) before a motor act even without consciousness of commanding it; 2) in situations that do not involve movement, such as decision-making in mental arithmetic [75], and 3) in externally triggered action [76]. Our concern here is not so much with the interpretation but with the development and time course of the processes associated with intentional inhibition.

Only a few studies have investigated the neural mechanisms of intentional inhibition using EEG [20, 21, 77–80]. Tasks in those studies were suboptimal in terms of 1) the choice between acting and withholding is relatively arbitrary; 2) pre-decision on whether and when to inhibit cannot be excluded; 3) perhaps tapping into selective choice rather than inhibition, especially when equiprobable go and no-go trials are used [77, 78]. Thus, the underlying mechanism might entail not only intentional inhibition but be confounded by other components. The Chasing Memo task remedies these limitations, at least to some extent. A further departure from some previous studies was that components that are closely related to stimulus-driven inhibition, such as N2/P3 [81] were not analyzed. First, for intentional inhibition we focused on neural activities preceding rather than after intentional inhibition, as 1) this can help predict when intentional inhibition is likely to happen; 2) for voluntarily chosen action/inhibition, nearly all cognitive processes happened before execution of the action; 3) there is no external stop-signal to be time-locked to, which makes the comparison with cued-inhibition on N2/P3 less relevant. Second, N2/P3 comprises a complex of well-known EEG component that is typically associated with cued-inhibition. Since the focus here is not on replicating previous findings of cued inhibition but on exploring the neural activities relevant to intentional inhibition as compared to cued inhibition, and since no N2/P3 could be expected (or indeed observed) for intentional inhibition, our focus was on the RP rather than the N2/P3 complex.

In Experiment II, we adopted a double-blind, within-subject cross-over design with participants tested once under alcohol and once under placebo. Brain activities were recorded with EEG when they were performing the Chasing Memo task. We hypothesized that the RP appears only in the intentional inhibition condition but not in the stimulus-driven inhibition condition. Second, in line with Experiment I, acute alcohol use may incur either stopping impulsivity or waiting impulsivity in disengaging from the action. The finding reported by Libet and colleagues (1983) suggests that the RP is positively associated with cognitive engagement and effort with respect to the impending movement [38]. The more the participant thinks about the action, the earlier and larger is the RP [82]. Thus, in the case of stopping impulsivity, the activation required to implement and set off the disengagement from action may take longer to build up, and may require higher criterion levels of such activation; hence, acute alcohol should result in an earlier onset of the RP and a larger area between onset and peak (area under the curve, AUC). Likewise, in the case of alcohol-induced waiting impulsivity, a RP onset that occurs at a relatively brief interval relative to the time of disengagement and a smaller AUC of the RP should be expected. As exploratory measures of secondary interest, we also compute peak amplitudes, and RP interval (from onset latency to peak latency).

### Methods^6^

#### Participants

Twenty right-handed male adults independent from Experiment I participated in this study, with an age range of 21 to 28 years old (*M* = 24.6, *SD* = 2.3). Participants were psychology students recruited from the local campus. According to self-report, they had a normal or corrected-to-normal vision, were subjectively in good health, and had no history of head injuries or neurological or psychiatric disorders, including obesity and anorexia. Although all participants were light to moderate drinkers in daily life, they did not engage in excessive consumption of alcohol or drugs and were not addicted to alcohol or other drugs. The study was approved by the local ethics committee and complied with the declaration of Helsinki, relevant laws, and institutional guidelines.

#### Alcohol administration

Drinks were orange juice mixed with either 40% alcoholic vodka or water. The amount of vodka was calculated depending on the participants’ body weight to obtain blood alcohol levels (BAC) of 0.05%. The mixture was divided into three equal portions. Two of the drinks were served with 5 min apart, prior to commencing the task. Up to 3 min was allowed for drinking each unit, followed by 2 min of mouth-wash to remove the residual alcohol in the mouth. About 40 minutes after the second drink, the third booster drink was served to reduce noise due to measuring during the ascending versus descending limbs of the blood alcohol curve [83]. To enhance the alcohol taste, all the drinks had a lemon soaked in vodka, and the glass in which drinks were served was sprayed with vodka beforehand. To mask the alcohol taste all drinks contained three drops of Tabasco sauce (McIIhenny Co., USA) [84]. Thus in either condition, participants were unable to distinguish alcohol from placebo on the basis of smell or taste.

#### Procedure

Each participant performed the experiment twice with 2 to 7 days in between. They were informed that they would receive a low dose and a high dose of alcohol for two sessions. This assured the presence of expectancy effects in both sessions. In one test session, they received alcoholic drinks; in the other session, they were actually given placebo drinks. Sessions took place between 12:00 and 6:00 p.m. at fixed times across conditions per individual. The order of experimental conditions was randomized in a double-blind cross-over design. Breath alcohol concentration (BrAC) was measured using the Lion alcolmeter® SD-400 and registered at four times during each session (i.e., baseline, after the first two drinks, pre and post the third drink, and by the end of the computer task). BrAC was measured by a second experimenter, who also prepared the beverages, with the primary experimenter always remaining blind to alcohol conditions and BrAC. A short manipulation check interview was performed at the end of each session to make sure participants are aware of the alcohol content of the drink. Participants provided informed consent prior to participation and were compensated with 20 euro for participation, plus a maximum of 5 euro extra depending on their performance. They were allowed to leave the lab only when their BrAC value was below 0.02% in the drink session.

#### Chasing memo task

Task details were identical to those reported in Experiment I, except for a color adjustment (the circle that turned from orange to blue and vice-versa in Experiment I turned from red to green and vice-versa in Experiment II), to better mimic traffic light-related associations with stopping and going. A practice stage and a test stage containing three free blocks and three cued blocks were included.

#### EEG data recording and preprocessing

Continuous EEG data were recorded using the BioSemi ActiveTwo system [85] and sampled at 2048 Hz. Recordings were taken from 64 scalp electrodes placed on the basis of the 10/20 system, and two additional electrodes were placed on the left and right mastoids. In addition, four electrodes were used to measure horizontal and vertical eye movements. In the BioSemi system, the ground electrode is formed by the Common Mode Sense active electrode and the Driven Right Leg passive electrode.

All EEG data were preprocessed and analyzed with EEGLAB v.13.5.4b [86], an open source toolbox for Matlab and Brain Vision Analyzer 2.0. Four participants were excluded from the analysis. One participant always disengaged when the star was presented on the screen (contrary to instructions). Three other participants had to be discarded due to technical malfunctions. Therefore data analyses were based on the remaining 16 participants. Data were imported to EEGLAB with average mastoids as the reference. Then, downsampled to 512 Hz and digitally filtered using a FIR filter (high pass 0.016 Hz and low pass 70 Hz, with an additional 50 Hz notch-filter). The EEG traces were then segmented into epochs ranging from − 3000 to 1000 ms (− 3000 to − 2500 was used for baseline correction), time-locked to the last disengagement moment before the completion of a trial.

Before artifact removal, trials in the free condition without a valid voluntary disengagement (i.e., disengagement occurring within 2 s following the bonus star, after which the trial ended automatically) were discarded, as intentional inhibition cannot be verified in these cases. Subsequently, artifact removal was accomplished in two steps. The first step consisted of visual inspection of the epochs to remove those containing non-stereotyped artifacts such as head or muscle movements, on the basis of manual and semi-automatic artifact detection (50 μV/ms maximal allowed voltage step, 150 μV maximal allowed difference of values in the epoch). This resulted in averages (SD) of 45.06 (7.30), 44.56 (9.37), 53.0 (7.47), and 52.94 (7.45) trials for alcohol/free, placebo/free, alcohol/cued, and placebo/cued conditions, respectively. The number of epochs removed never exceeded 25%. Secondly, an independent component analysis (ICA) was performed using the ‘runica’ algorithm available in EEGLAB [87]. The extended option was used that implements a version of the infomax ICA algorithm [88] resulting in better detection of sources with sub-Gaussian distribution, such as line current artifacts and slow activity. Then we applied the algorithm ADJUST that automatically identifies artefactual independent components by combing stereotyped artifact-specific spatial and temporal features [89]. ADJUST is optimized to capture blinks, eye movements, and generic discontinuities and has been validated on real data. After exclusion of artefactual components the data were reconstructed based on an average (SD) of 55.57 (3.72), 57.69 (2.91), 56.75 (3.15), and 58.75 (3.21) ICA components in the alcohol/free, placebo/free, alcohol/cued, and placebo/cued conditions, respectively. The number of independent components removed did not exceed 14% of the total in any of the conditions.

Afterward, data were re-referenced using the current source density (CSD) transformation [90] as implemented in Brain Vision Analyzer [91] (with the parameters degree of spline = 4; maximum degrees the Legendre polynomial = 15). The CSD transformation uses surface Laplacian computation to provide a reference-free estimate of the local radial current density rather than distant/deep (neural) sources [92, 93]. A major advantage is that CSD leads to the enhanced spatial precision of the recorded EEG activity [94, 95] and thus acts as a spatial filter. Finally, epochs were averaged for each participant and experimental condition for further statistical analysis. Previous literature indicates that the supplementary motor areas contribute considerably to the generation of the RP. Although some studies have analyzed the RP based on a pool of electrodes surrounding FCz, several studies suggest that the activity of these regions is best captured by electrode FCz [96, 97], especially after CSD transformation. This was confirmed by visual inspection for each participant. Statistical analyses were therefore conducted only on this electrode.

#### Data preparation and statistical analysis

##### Task performance

The calculations for median Engage RT, Disengage RT and W-interval were the same as in Experiment I. Engage RTs of less than 100 ms were removed, resulting in 916 (95%), 885 (92%), 892 (93%), and 931 (97%) trials for alcohol/free, placebo/free, alcohol/cued, and placebo/cued conditions, respectively. For Disengage RT in the free condition, if the participant did not voluntarily disengage within the provided time, that trial was removed. This resulted in 788 (82%) trials for the alcohol condition and 836 (87%) trials for the placebo condition. Independent t-tests were performed to compare performance under placebo and alcohol conditions for each of these dependent variables.

##### EEG

Four indices extracted from the ERP topographic plots were analyzed, including RP onset latency, RP peak amplitude, AUC, and RP build-up interval (from onset latency to peak latency). For RP onset latency, since automated algorithms failed to yield consistent and robust latencies for most participants, three authors (YL, GFG, & RR) independently judged the EEG time courses for each individual trial, while they remained blind to Inhibition Category. The raters hand-picked (through computer-aided scrolling procedures) the RP onset as the moment in time (in ms) when the signal began to deviate and showed a steady switch towards the negative direction. The inter-rater reliability calculated by intraclass correlation was 0.96, which indicated high consistency among raters. AUC was quantified as the total surface in the time window between onset latency and peak latency, using the R package ‘stats’ (version 3.3.0) [98]. A two-way within-subject repeated-measures ANOVA was implemented with Alcohol (alcohol/placebo) and Inhibition Category (free/cued) as factors.

##### Conventional and Bayesian-based analysis

As in Experiment I, we did both conventional and Bayesian-based paired *t*-test and repeated-measures ANOVA analysis for the main dependent variables. Bayesian repeated-measures ANOVA compares all the models against the null model. BF was provided every time a main factor or interaction was added to the model, allowing us to establish how each main factor and the interaction contributed to the model.

### Results

#### BrAC

The descriptive values at each reading can be found in Additional file 1. In brief, BrAC peaked after the third drink, with a mean value of 0.06% and a standard deviation of 0.10.

#### Task performance

In brief, acute alcohol use did not exert meaningful effects on Engage RT/Disengage RT in either the cued or free condition. Similarly, alcohol did not influence timing accuracy and W-interval. More detailed information can be found in Additional file 1.

#### EEG

##### RP onset latency

**Fig. 2:**
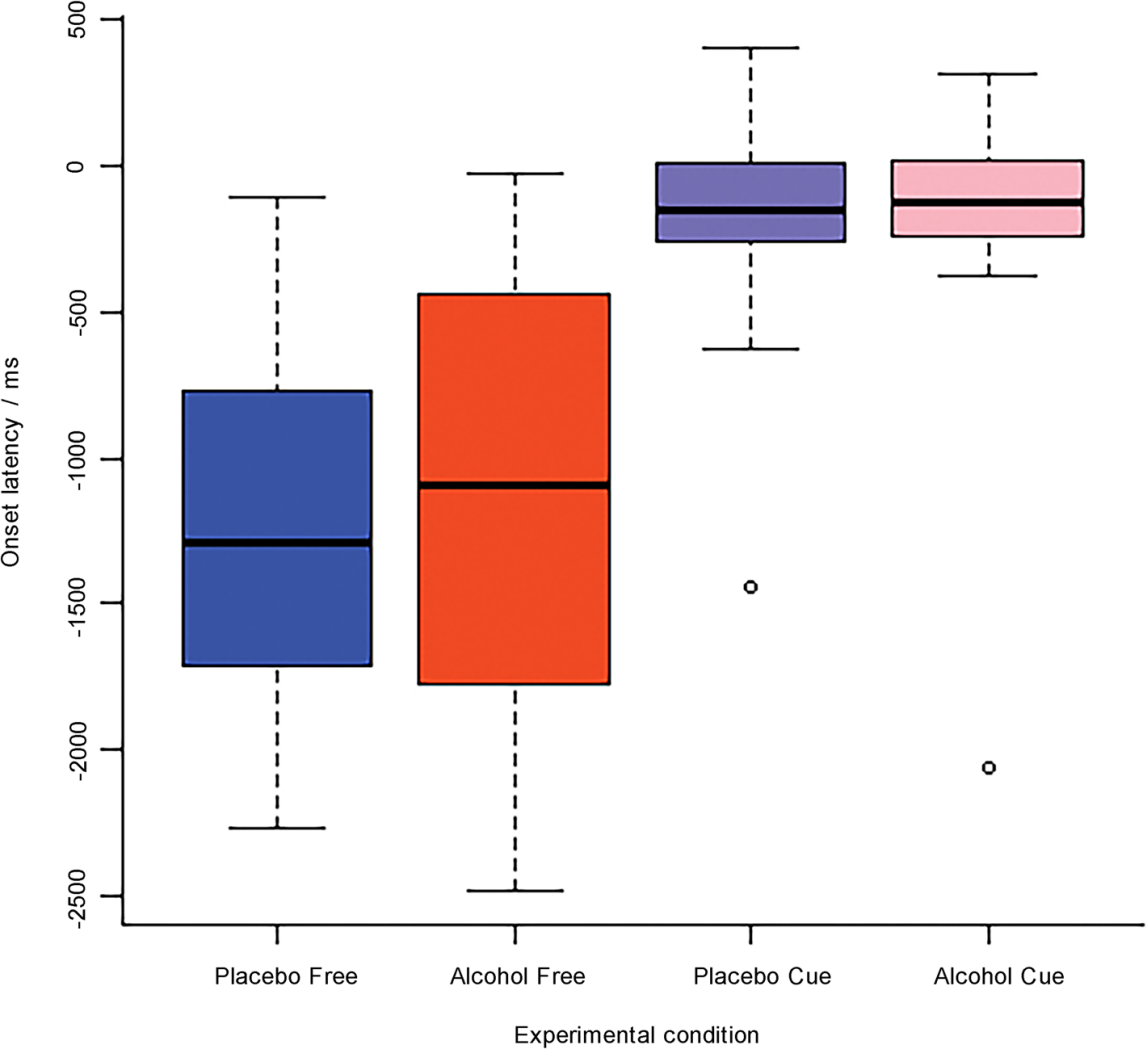
Boxplot of the onset latency (in ms) of the Readiness Potential per group: Alcohol (alcohol vs. placebo) × Inhibition Category (cued vs. free). Only a main effect of Inhibition Category is observed

**Fig. 3:**
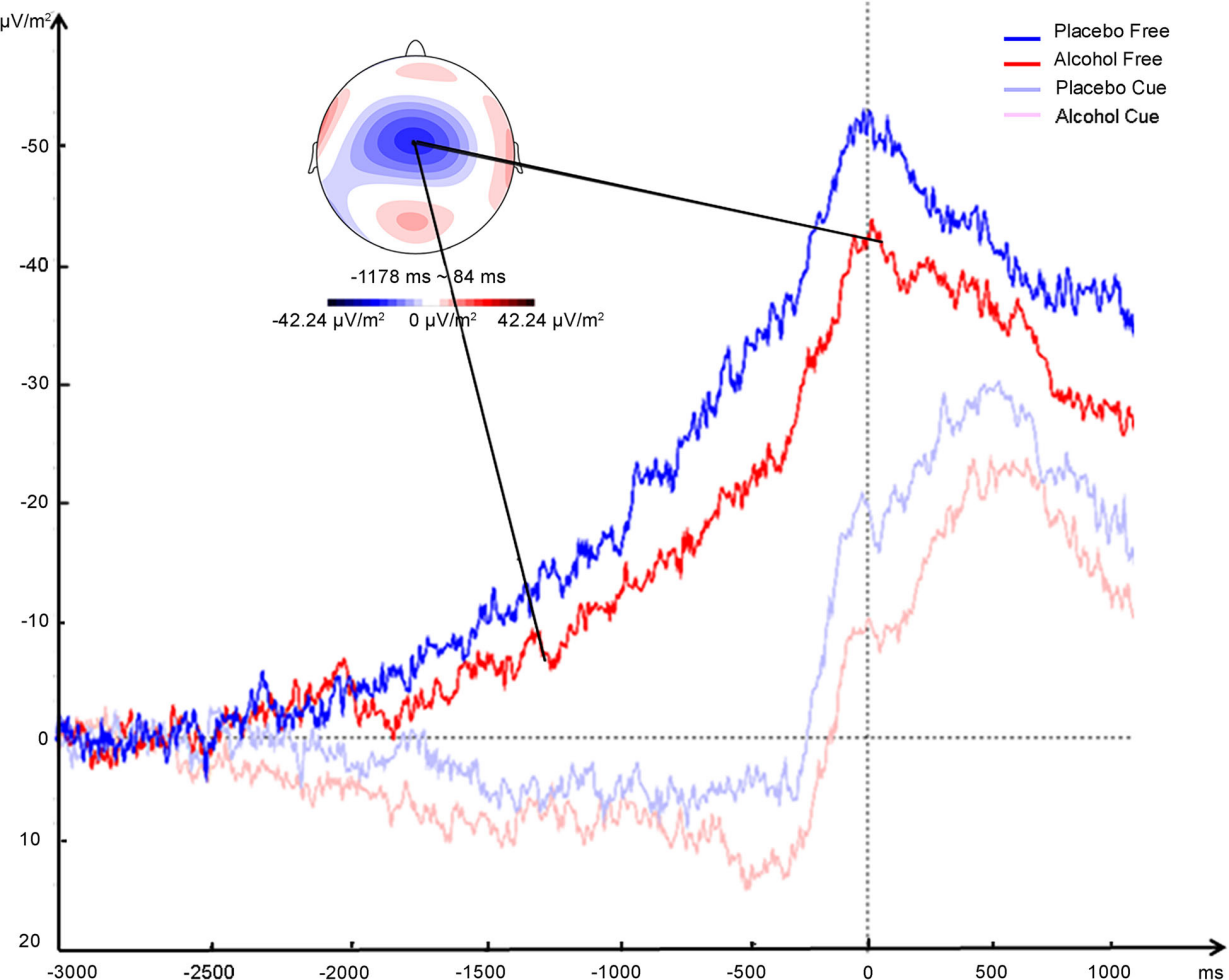
Surface Laplacians over electrode FCz for the free and cued inhibition under alcohol or placebo conditions. Traces are time-locked to disengagement time (time 0). The scalp map shows mean activity in the time window of the RP, as defined by RP onset and peak amplitude for the free inhibition condition under alcohol. Electrode FCz is marked in the scalp maps (black dot)

Repeated-measures ANOVA confirmed that the main effect of Inhibition Category was significant (*F* (1, 15) = 46.89, *p* < 0.001, *η*^*2*^ = 0.70), with much earlier onsets in the free condition (*M* = − 1229 ms, *SD* = 710) than in the cued condition (*M* = − 205 ms, *SD* = 464, see Figs. 2 and 3). The main effect of Alcohol was not significant (Alcohol: *M* = − 693 ms, *SD* = 839; Placebo: *M* = − 742 ms, *SD* = 745; *F* (1, 15) = 0.14, *p* = 0.72, *η*^*2*^ *=* 0.01). The interaction between Alcohol and Inhibition Category was also not significant (*F* (1, 15) = 0.20, *p* = 0.66). Bayesian repeated measures ANOVA showed that a model that contained only Inhibition Category provided a fit that was 3.6 times better than a model that added the factor Alcohol, and 10.3 times better than a model that further added the interaction effect. These results together confirmed the significant main effect of Inhibition Category in the absence of main and interaction effects of Alcohol.

##### AUC

**Fig. 4:**
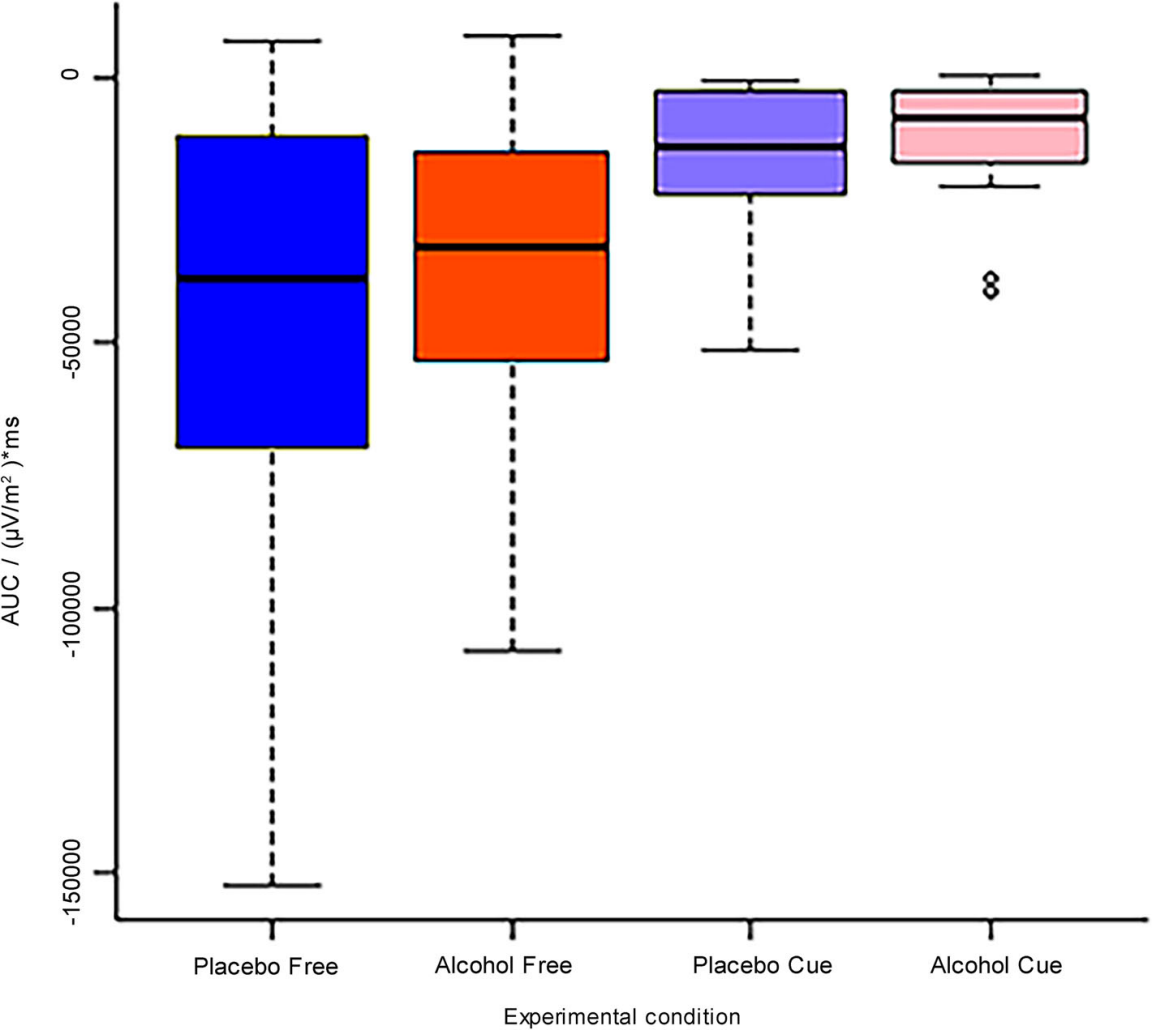
Boxplot of the area under the curve (AUC) (in (μV/m^2^)*ms) of the Readiness Potential per group: Alcohol (alcohol vs. placebo) × Inhibition Category (cued vs. free). Only a main effect of Inhibition Category is observed

Repeated-measures ANOVA confirmed a significant main effect of Inhibition Category (*F* (1, 15) *=* 21.04*, p <* 0.001*, η*^*2*^ *=* 0.58), with a much greater AUC in the free condition (*M* = − 40,563 (μV/m^2^)* ms, *SD* = 37,332) than in the cued condition (*M* = − 13,348 (μV/m^2^)* ms, *SD* = 13,815, Fig. 4). Although the AUC appeared reduced under alcohol compared to placebo, the main effect of Alcohol failed to obtain significance (Alcohol: *M* = − 23,323 (μV/m^2^)* ms, *SD* = 25,692; Placebo: *M* = − 30,588 (μV/m^2^)* ms, *SD* = 35,771; *F* (1, 15) *=* 1.22, *p =* 0.29, *η*^*2*^ = 0.08)*.* The interaction between Alcohol and Inhibition Category was not significant (*F* (1, 15) *=* 0.29, *p =* 0.60)*.* Bayesian repeated measures ANOVA showed that a model that contained only Inhibition Category in the model provided a fit that was 2.3 times better than model that added the factor Alcohol and 5.8 times better than a model that further added the interaction effect. These results together confirmed the significant main effect of Inhibition Category in the absence of main and interaction effects of Alcohol.

##### Summary of EEG results

Since the results of the analyses on RP peak amplitude and build-up interval were highly redundant to those of AUC, these results can be found in Additional file 1. In general, the four ERP indices provided a consistent pattern of the RP that was influenced considerably by the factor Inhibition Category but was not influenced by the factor Alcohol. Under free inhibition, the RP began to develop almost 1000 ms earlier than under cued inhibition. Also, under free inhibition, the RP reached higher peak amplitudes than under cued inhibition. Accordingly, the AUC is larger for free than for cued inhibition. Generally speaking, only under free inhibition condition, there was a clear RP before disengagement. But these effects were not impacted by the acute effects of alcohol.

### Discussion

In this experiment, we tested how moderate acute alcohol use influences intentional inhibition and stimulus-driven inhibition, at behavioral as well as neural levels. RP developed over the frontocentral cortex about 1200 ms before intentional inhibition was effectuated but not before stimulus-driven inhibition. It turned out that alcohol administration had hardly any effect, either behaviorally or on neural correlates of intentional inhibition and stimulus-driven inhibition. These null-findings were corroborated by Bayesian analyses that confirmed there was stronger evidence for the null hypothesis than for the alternative hypothesis.

#### Stimulus-driven inhibition

In contrast to previous findings on impaired stimulus-driven inhibition after alcohol intake [67–71, 99, 100], no alcohol effects were observed on stimulus-driven inhibition as measured in the Chasing Memo task. Since the present study did not include a SST or a GNG task, we cannot tell whether the lack of effects is specific to the Chasing Memo task or pertains to our alcohol manipulation in the present sample.

A number of potential reasons may explain the discrepancy between the present and previous findings in the literature. First, the doses of alcohol administered in the present study may have been too low to produce manifest alcohol effects. Previous studies have demonstrated effects on ERP components under comparable alcohol doses and sample size [101]. But compared with the flanker task they used, disengaging from visuomotor tracking in the Chasing Memo task was relatively easy. And it has been pointed out that the easier the task, the more alcohol is needed to cause performance impairments [17]. Our conclusions cannot be generalized to the full range of acute intoxication. Second, alcohol effects may be confounded with individual differences in alcohol expectancy effects [102]. For instance, it has been observed that those who expect less alcohol-induced impairment indeed displayed less impairment, irrespective of actual consumption [103–105]. Without an additional control group (participants who do not get any alcohol, and who know so) in the current study, it is difficult to distinguish between expectancy and pharmacological effects of alcohol [106]. Third, although alcohol intake resulted in similar BACs across participants, there might still exist non-trivial individual differences in the actual impairment instilled by alcohol [106].

### Intentional inhibition

Previous studies did not examine the EEG effects of alcohol on intentional inhibition. We observed no effects, neither from the perspective of stopping impulsivity nor waiting impulsivity. The factors that were discussed that potentially play a role in the absence of alcohol effects on stimulus-driven inhibition may also pertain to intentional inhibition. In particular, individual differences in the actual impairment caused by alcohol [106]. Indeed, individual data in our study showed that roughly half of the participants had earlier RP onsets under alcohol, while the opposite pattern was observed among the other half. Furthermore, a true effect might have been missed due to low power from the small sample size. Future studies may explore such individual differences more systematically and recruit a larger sample. Second, the requirement to report the W-moment might interfere with the main task at hand (continue/disengage tracking). This process required attention shifting (i.e., have a glance of the counter) and working memory storage (i.e., keep this number in memory). Meanwhile, the reliability of reported W-moment has been questioned [107]. Therefore, future studies not focused on consciousness may consider discarding this element.

## General discussion

Many studies have investigated the relationship between alcohol use and inhibition, but all previous studies focused on stimulus-driven inhibition, typically tested with varieties of the GNG and SST. Here, we expanded this focus by testing alcohol effects on intentional inhibition in two studies: focused on past-year risky drinking and short-term alcohol use respectively. Both intentional inhibition and stimulus-driven inhibition were tested. We found no relationship between past-year moderate recreational alcohol use with both types of inhibition and no differences related to moderate acute alcohol administration. The main finding was that the RP showed an earlier onset and higher peak values for intentional compared to stimulus-driven inhibition, independent of alcohol administration.

Regarding stimulus-driven inhibition, its null association with past-year alcohol use is to some extent in correspondence with the literature. Presumably, a threshold effect rather than a linear relationship exists between typical alcohol use and response inhibition. That is, only when the accumulated alcohol consumption surpassed a certain threshold or a diagnosis of AUD is confirmed, long-term alcohol use is accompanied by impaired inhibition [108–111]. Accordingly, our conclusions cannot be readily generalized to the population with AUD. On the other hand, our lack of effects of acute alcohol use on stimulus-driven inhibition is more at odds with previous research. A study by Marczinski et al. (2005) using a cued GNG showed impaired inhibition of a button press (i.e., a discrete motor response) under the influence of alcohol [112]. However, alcohol did not influence inhibition performance if participants had to *release* instead of *press* a button (i.e., a continuous movement). This latter response type seems to resemble the ongoing tracking movements in the Chasing Memo task. The employment of discrete go responses can explain why the acute effects of alcohol are frequently reported on GNG and SST [67, 69] but not in our task.

Regarding intentional inhibition, our studies represent the first exploration of a potential link with alcohol use and misuse. Neither effects of trait drinking patterns (social/problematic) nor acute alcohol effects were observed. This negative finding coincides with a recent finding in Parkinson patients. Three groups of participants (healthy control, Parkinson with and without impulsive-compulsive behaviors) did not differ on intentional inhibition performance measured by the Marble Task [113]. This suggests that populations that typically show comorbid impaired reactive inhibition, such as Parkinson disease, ADHD, and substance use disorder, can still keep intentional inhibition capability intact.

At the neural level, a slow negative potential appeared 1200 ms exclusively before intentional inhibition, which provides evidence that the RP also reflects the preparation of stopping a motor action. Together with the evidence that the RP develops prior to the process irrelevant to action [114–116] and its amplitude is influenced by the degree of intentionality [117–119], it is concluded that RP reflects neural processes related to intention formation rather than motor preparation [114, 120, 121]. This can also be interesting in relation to the current discussion on the brain disease model of addiction [122] and with respect to the question if long-term alcohol-dependent patients show problems in intention formation and/or execution.

We acknowledge a number of limitations of our study. First, in the Chasing Memo task, participants were obliged to disengage on all free trials. The moment of disengagement was ‘at will’, but disengagement at any point during a free trial was mandatory rather than voluntary. If we had added the ‘whether’ option and let participants determine more freely if and when to disengage, alcohol might still influence decisional aspects of intentional inhibition [123]. Just like the priming effect of alcohol, preload drinking promoted loss of control over further drinking behavior [17]. In that way, acute alcohol use should increase the *probability* of accepting another beer rather than *when* you accept it. We are currently exploring intentional inhibition and effects of alcohol in a modified version of the Chasing Memo task with a ‘whether’ option added. Second, gender was disproportionally distributed in both experiments. In Experiment I, there was more females than males. We, therefore, added gender as a covariate in the main analyses and confirmed its null effect. Experiment II included only male participants given sex differences in metabolic alcohol processing. We cannot be sure if the current findings generalize to females. Future studies might aim at more gender-balanced samples. Third, our sample size in Experiment II is relatively small, but studies with a similar topic and study design confirmed its power [77]. Fourth, there is room for alcohol administration and placebo conditions to be improved, given that although all participants reported they received alcohol in the placebo condition, the amount is less than that in the alcohol condition; the experimenter blind to alcohol condition may interact with participants differently in two conditions (alcohol/placebo) due to the participants’ status (drunk/sober). We acknowledge this as a potential shortcoming, although these are common issues in this field, and generally not considered overly detrimental to interpretation.

We end by providing a few suggestions for future research into this field. First, the target population may include heavier binge drinkers and/or alcohol-dependent patients. It has been shown that impairments in inhibitory control after a moderate dose of alcohol are more pronounced in binge drinkers than in non-binge drinker subjects [124]. This might help explain that when these individuals become intoxicated, they are less able to refrain from the impulse or desire to consume more alcohol, leading to further binge drinking. Further, one might employ intravenous alcohol administration to keep the BAC at a steady level for a prolonged time [125]. This can help control the acute tolerance effect of alcohol (reduced impairment at a given BAC on the descending limb) [126]. In addition, alcohol-related cues may be embedded in the task as they are more salient for heavy drinkers (compared to light drinkers) and can impact on inhibitory processes [127, 128]. Also, it is interesting to explore whether only a subgroup of the drinkers with specific drinking patterns and personalities show intentional inhibition deficits.

### Conclusion

This is the first empirical study on the role of intentional inhibition in relation to alcohol use. In two experiments, we found that both past-year risky drinking and moderate acute alcohol did not affect intentional inhibition, suggesting that alcohol does not moderate the ability to stop at will in the present study. Factors that might explain these null findings, such as the lifetime amount of alcohol used, alcohol administration dosage, and research paradigms were discussed. Caution should be taken when extending these conclusions to AUD populations and higher intoxication levels (e.g., 0.08%). In addition, we found an event-related brain potential, the readiness potential (RP), that appeared 1.2 s before the intentional inhibition of action. No RP was visible before stimulus-driven inhibition. This indicates that the RP might reflect the formation of an intention in general rather than only signifying motor preparation.

## Supplementary information

**Additional file 1:** Experiment I: 1) reliability of the questionnaires used; 2) results of the two computer tasks when AUDIT-C was used; 3) results of the stop-signal task for the 86 participants sample. Experiment II: 1) BrAC values at each reading; 2) behavioral findings; 3) EEG results for RP peak amplitude and RP build-up interval.

## Data Availability

The datasets generated during and/or analyzed during the current study are available from the corresponding author on reasonable request.

## Abbreviations

ADHD: Attention-Deficit/Hyperactivity Disorder
AUC: Area Under Curve
AUD: Alcohol Use Disorder
AUDIT: Alcohol Use Disorder Identification Test
BAC: Blood Alcohol Concentration
BF: Bayesian Factor
BIS: Barratt Impulsiveness Scale
BrAC: Breath Alcohol Concentration
CSD: Current Source Density
CUDIT-R: Cannabis Use Disorder Identification Test-Revised
DII: Dickman’s Impulsivity Inventory
EEG: Electroencephalography
ERP: Event-Related Potential
FCz: a channel in the 10–20 EEG system GNG Go/No-Go Task
go RT: Go Reaction Time
ICA: Independent Component Analysis
M: mean value
mFTQ: Modified Version of the Fagerström Tolerance Questionnaire
N2, P3: event-related potential components
RP: Readiness Potential
SD: Standard Deviation
SSD: Stop Signal Delay
SSRT: Stop Signal Reaction Time
SST: Stop Signal Task
SUD: Substance Use Disorder
VIF: Variance Inflation Factors

## Footnotes

1 This was part of YL’s PhD thesis [37].

2 For computers with incorrect refresh rate settings, Memo moved at twice of the speed as planned, which made the moment of disengagement incomparable.

3 Participants were not dichotomized into light and heavy drinkers during recruitment and data analysis stage as there was individual variance of alcohol consumption in these broad groups and artificial dichotomization reduces the power to detect subtle individual differences [44]. In addition, we replicated these analyses by replacing AUDIT total score by AUDIT-C (the first three items of AUDIT), which is not limited to the past 12 months.

4 Other substances use were not added as a covariate as they were highly correlated with the AUDIT score (see Table 2).

5 We only expect a small to moderate relationship between SSRT and disengage RT as intentional inhibition engaged additional neural activation albeit common inhibition network with stimulus-driven inhibition [22].

6 This was part of YL’s PhD thesis [37].
