# Supplementary material for: “Free won’t” after a beer or two: chronic and acute effects of alcohol on neural and behavioral indices of intentional inhibition
Source: BMC Psychol. 2020 Jan 7;8:2. doi: 10.1186/s40359-019-0367-z (PMC6947965; doi:10.1186/s40359-019-0367-z)
Supplement: Supplementary file 1 — Additional file 1: Experiment I: 1) reliability of the questionnaires used; 2) results of the two computer tasks when AUDIT-C was used; 3) results of the stop-signal task for the 86 participants sample. Experiment II: 1) BrAC values at each reading; 2) behavioral findings; 3) EEG results for RP peak amplitude and RP build-up interval. [file 40359_2019_367_MOESM1_ESM.docx]

**Additional file 1**

**[Title]**

“Free won't” after a beer or two: Chronic and acute effects of alcohol on neural and behavioral indices of intentional inhibition

**[Journal name]**

BMC psychology

**Experiment I**

**Reliability of questionnaires:**

AUDIT: The Dutch version was approved to be a reliable instrument [1]. Cronbach’s alpha in the current study was 0.85.

The modified version of the fagerström tolerance questionnaire (mFTQ): Diagnostic score as follows: 0-2 indicates no dependence, 3-5 indicates moderate dependence and 6-9 indicates substantial dependence. The Dutch version had test-retest reliability ranging from 0.70 to 0.91 for different sex [2].

The cannabis use disorder identification test revised (CUDIT-R): The CUDIT-R was shown to be a reliable and valid screening test [3]. A cut-off score of 8 or higher is used to indicate a reasonable suspicion of problematic cannabis use.

Core alcohol and drug survey (CORE): the eleven substances include alcohol, tobacco, marijuana, cocaine, amphetamines, sedatives, hallucinogens, opiates, inhalants, designer drugs, and steroids .

Barratt Impulsiveness Scale -11: Cronbach’s alpha for the Dutch version is 0.81 [4].

Dickman’s Impulsivity Inventory: Cronbach’s alpha coefficient of the Dutch version was 0.84 for the dysfunctional dimension and 0.76 for the functional dimension [5].

**Results when AUDIT-C is used**

**Chasing memo task**

The linear regression model for Engage RT was not significant (*F* (3, 116) = 0.81, *p =* 0.49), with a *R^2^* of 0.021. None of the explanatory variables significantly predicted Engage RT (AUDIT-C: *β =*0.07*, p =* 0.46; Inhibition Category: *β =* -0.02*, p =* 0.84; gender: *β =* -0.12*, p =* 0.18). Bayesian linear regression showed that the null model provided a fit that was 2.2 times better than the model that added the factor gender, 3.9 times better than the model that added AUDIT-C and 5.1 times better than the model that added Inhibition Category.

The linear regression model for Disengage RT was significant (*F* (3, 116) = 93.72*,* *p* < 0.01), with a *R^2^* of 0.71. Inhibition Category significantly predicted Disengage RT (*β =* 0.84*,* *p* < 0.01). Disengage RT was much longer in the free condition than in the stimulus-driven inhibition (8662 ms vs. 749 ms). Neither AUDIT-C (*β =* -0.04*, p =* 0.46) nor gender (*β =* 0.06*, p =* 0.26) predicted Disengage RT. Bayes factor analysis confirmed this by showing that the model with factor Inhibition Category provided a fit that was 7.1 times and 10.1 times better than the model that further added factor Gender and AUDIT-C, respectively.

Past-year alcohol consumption (i.e., AUDIT-C) is not associated with timing accuracy alteration (*r* = -0.21, *p* = 0.10, BF_01_ = 1.64). The linear regression model for W-interval was not significant (*F* (2, 57) = 0.25, *p =* 0.78), with a *R^2^* of 0.009. None of the explanatory variables significantly predicted W-interval (AUDIT-C: *β =* -0.06*, p =* 0.64; timing accuracy: *β =* -0.08*, p =* 0.54). Bayes factor analysis confirmed this by showing that the null model provided a fit that was 3.4 times, and 3.6 times better than the model that added the factor Timing Accuracy and AUDIT-C, respectively.

**SST (N=60)**

The linear regression model for SSRT was not significant (*F* (2, 57) = 0.42, *p =* 0.66), with a *R^2^* of 0.01. None of the explanatory variables significantly predicted SSRT (AUDIT-C: *β =* 0.10*, p =* 0.46; gender: *β =* 0.07*, p =* 0.58). Bayes factor analysis confirmed this by showing that the null model provided a fit that was 3.4 times better than the model that added the factor AUDIT-C or Gender. The linear regression model for go RT was not significant either (*F* (2, 57) = 2.27, *p =* 0.11), with a *R^2^* of 0.07. AUDIT-C was a significant predictor of go RT (*β =* -0.26*, p =* 0.05), indicating the higher the AUDIT-C score the shorter the go RT. Gender was not a strong predictor of go RT (*β =* -0.08*, p =* 0.52). Bayes factor analysis indicated anecdotal evidence for the effect of AUDIT-C, i.e., adding it to the model was just 1.5 times better than the null model. And the fitness of the null model is 3.3 times better than adding factor Gender.

**Results for the stop-signal task for the 86 participants sample**

The linear regression model for SSRT was not significant (*F* (2, 83) = 0.57, *p =* 0.57), with an *R^2^* of 0.014. None of the explanatory variables significantly predicted SSRT (AUDIT: *β =* 0.07*, p =*0.52; gender: *β =* 0.09*, p =*0.41). Bayes factor analysis confirmed this by indicated evidence for a lack of effect of AUDIT (BF_01_ = 3.28) and gender (BF_01_ = 2.89).

The linear regression model for go RT was not significant (*F* (2, 83) = 0.17, *p =* 0.17), with an *R^2^* of 0.04. Neither AUDIT (*β =* -0.21*, p =*0.06) nor gender (*β =* 0.025*, p =*0.82) was a significant predictor of go RT. Bayes factor analysis indicated anecdotal evidence for the effect of AUDIT (BF_10_ = 1.36) and a lack of evidence for the effect of gender (BF_01_ = 4564).

**Experiment II**

**BrAC values at each reading**

The BAC measured 5 minutes after finishing the second drink (BrAC_1_) ranged from 0.014% to 0.087% BAC (*M* = 0.05%, *SD* = 0.09). BrAC_2_, measured before the third drink ranged from 0.018% to 0.064% (*M* = 0.039%, *SD* = 0.05). BrAC_3_, measured after completion of the third drink, ranged from 0.025% to 0.094% (*M* = 0.062%, *SD* = 0.10). BrAC_4_, measured by the end of the task, ranged from 0.009% to 0.073% (*M* = 0.046%, *SD* = 0.06).

**Behavioral finding**s

***Engage RT***

Basic response speed (i.e. Engage RT in the cued condition) was similar across alcohol (*M* = 431 ms, *SD* = 75.6) and placebo conditions (*M* = 411 ms, *SD* = 78). This 20 ms difference was statistically reliable (*t*(15) = 2.18, *p* = 0.05, *d* = 0.55). However the Bayesian paired t-test provided only anecdotal support in favor of for the alternative hypothesis (*BF_10_* = 1.62).

In the free condition, participants started tracking a bit slower than in the cued condition. Engage RT did not differ between conditions (alcohol: *M* = 524 ms, *SD* = 263.3; placebo: *M =* 551 ms, *SD* = 299; *t*(15) = 0.89, *p* = 0.39). A Bayesian *t*-test provided anecdotal to moderate evidence for the null hypothesis (*BF_01_* = 2.78).

To sum up, compared to placebo, acute alcohol use did not exert meaningful effects on Engage RT in either the cued or free condition.

#### Disengage RT

#### In the cued condition, participants stopped tracking within one second following the external stop signal. Participants appeared to be slower to disengage under alcohol than under placebo condition (M = 554 ms, SD = 162, vs. M = 517 ms, SD = 151 respectively), but this effect did not reach statistical significance (t(15) = 0.89, p = 0.39). This was confirmed by a Bayesian t-test (BF_01_ = 2.78), indicating marginal evidence in favor of the null hypothesis.

In the free condition, participants fully exploited the available time window to disengage from tracking, with Disengage RTs ranging between 4 and 20 s, averaging to 11-12 s. Participants appeared to be slightly slower to disengage under alcohol than under placebo (*M* = 11,738 ms, *SD* = 2,354, vs. *M* = 11,684 ms, *SD* = 2,223), but this effect did not approach statistical significance (*t*(15) = 0.09, *p* = 0.93), as confirmed by a Bayesian *t*-test (*BF_01_* = 3.9), indicating moderate support in favor of the null hypothesis.

Thus, compared to placebo, alcohol failed to exert meaningful effects on Disengage RT in either the cued or free conditions.

***Time reporting***

Timing accuracy was inferred by subtracting the reported time of stop-signal presentation from the actual presentation time in the cued condition. Alcohol (*M* = 380 ms, *SD* = 231) and placebo conditions (*M* = 436 ms, *SD* = 367) were associated with comparable of time estimations (*t*(15) = -0.99, *p* = 0.34, *d* = -0.25, *BF_10_* = 2.57), indicating that alcohol did not affect time estimation.

In the free condition, the W-interval amounted less than half a second. Though participants were faster to disengage once they felt the urge under alcohol (*M* = 195 ms, *SD* = 1,413) than under placebo (*M* = 470 ms, *SD* = 2,141), but this difference was not significant (*t*(15) = 0.43, *p* = 0.65), as confirmed by Bayesian analysis (*BF_01_* = 3.61).

**EEG results**

RP peak amplitude

Repeated-measures ANOVA confirmed a significant main effect of Inhibition category (*F*(1, 15) *=* 15.06*, p* < 0.001*, η² =* 0.50), with an increased peak amplitude in the free condition (*M* = -55.43 μV/m^2^, *SD* = 30.53) than for cued condition (*M* = -35.83 μV/m^2^, *SD* = 19.41). Although the peak amplitude appeared reduced under alcohol compared to placebo, the main effect of Alcohol failed to reach significance (Alcohol: *M* = -41.77 μV/m^2^, *SD* = 25.01; Placebo: *M* = -49.49 μV/m^2^, *SD* = 29.18; *F*(1, 15) *=* 1.84, *p =* 0.20)*.* The interaction between Alcohol and Inhibition category was not significant (*F*(1, 15) *=* 0.12, *p =* 0.74)*.* Bayesian repeated measures ANOVA showed that a model that contained only inhibition category in the model provided a fit that was 1.5 times better than model that added the factor Alcohol condition and 4.3 times better than a model that further added the interaction effect.

RP build-up interval

Repeated-measures ANOVA confirmed that the main effect of Inhibition category was significant (*F*(1, 15) *=* 42.66*, p <* 0.001*, η² =* 0.74), with a much slower build-up interval in the free condition (*M* = 1,353 ms, *SD* = 692) than in the cued condition (*M* = 594 ms, *SD* = 427). The main effect of Alcohol was not significant (Alcohol: *M* = 945 ms, *SD* = 702; Placebo: *M* = 1,002 ms, *SD* = 682; *F*(1, 15) *=* 0.24, *p =* 0.63). The interaction between Alcohol and Inhibition category was not significant (*F*(1, 15) *=* 0.60, *p =* 0.45)*.* Bayesian repeated measures ANOVA showed that a model that contained only Inhibition category provided a fit that was 3.5 times better than a model that added the factor Alcohol condition, and 7.3 times better than a model that further added the interaction effect.

**Reference**

1. Hildebrand M, Noteborn MGC. Exploration of the (interrater) reliability and latent factor structure of the Alcohol Use Disorders Identification Test (AUDIT) and the Drug Use Disorders Identification Test (DUDIT) in a sample of Dutch probationers. Subst Use Misuse. 2015;50:1294–306. <https://doi.org/10.3109/10826084.2014.998238>

2. Vink JM, Willemsen G, Beem AL, Boomsma DI. The Fagerström Test for Nicotine Dependence in a Dutch sample of daily smokers and ex-smokers. Addict Behav. 2005;30:575–9. <https://doi.org/10.1016/j.addbeh.2004.05.023>

3. Adamson SJ, Kay-Lambkin FJ, Baker AL, Lewin TJ, Thornton L, Kelly BJ, et al. An improved brief measure of cannabis misuse: The Cannabis Use Disorders Identification Test-Revised (CUDIT-R). Drug Alcohol Depend. 2010;110:137–43. <https://doi.org/10.1016/j.drugalcdep.2010.02.017>

4. Goudriaan AE, Oosterlaan J, De Beurs E, Van Den Brink W. The role of self-reported impulsivity and reward sensitivity versus neurocognitive measures of disinhibition and decision-making in the prediction of relapse in pathological gamblers. Psychol Med. 2008;38:41–50. <https://doi.org/10.1017/s0033291707000694>

5. Claes L, Vertommen H, Braspenning N. Psychometric properties of the Dickman impulsivity inventory. Pers Individ Dif. 2000;29:27–35. <https://doi.org/10.1016/s0191-8869(99)00172-5>
